# Multivariate linear QSPR/QSAR models: Rigorous evaluation of variable selection for PLS

**DOI:** 10.5936/csbj.201302007

**Published:** 2013-03-02

**Authors:** Kurt Varmuza, Peter Filzmoser, Matthias Dehmer

**Affiliations:** a Institute of Chemical Engineering, Vienna University of Technology, Austria; b Department of Statistics and Probability Theory, Vienna University of Technology, Austria; c Institute for Bioinformatics and Translational Research, UMIT - The Health and Life Sciences University, Hall in Tyrol, Austria; # These authors contributed equally to this work

**Keywords:** molecular descriptors, PLS, variable selection, cross validation, software R

## Abstract

Basic chemometric methods for making empirical regression models for QSPR/QSAR are briefly described from a user's point of view. Emphasis is given to PLS regression, simple variable selection and a careful and cautious evaluation of the performance of PLS models by repeated double cross validation (rdCV). A demonstration example is worked out for QSPR models that predict gas chromatographic retention indices (values between 197 and 504 units) of 209 polycyclic aromatic compounds (PAC) from molecular descriptors generated by *Dragon* software. Most favorable models were obtained from data sets containing also descriptors from 3D structures with all H-atoms (computed by *Corina* software), using stepwise variable selection (reducing 2688 descriptors to a subset of 22). The final QSPR model has typical prediction errors for the retention index of ±12 units (95% tolerance interval, for test set objects). Programs and data are provided as supplementary material for the open source *R* software environment.

## Introduction

Properties or activities of chemical compounds can be estimated by regression models that relate a set of molecular descriptors with the property or activity. A large group of molecular descriptors [1] comprise the so-called topological (or structural) measures, which are based on structural features of the molecular structures under consideration. This group can be further divided into several categories such as information-theoretic indices, eigenvalue-based indices, matrix-based indices, distance-based measures, and so forth. But this does not mean that a particular index can be always classified uniquely into a given category. In fact, many indices exist which are based on several categories, e.g., eigenvalue or distance-based indices based on Shannon's entropy.

The above mentioned quantitative structure-property or structure-activity relationships (QSPR/QSAR) [2] can often be successfully described by linear models of the form

$$\hat{y}=b_{1}x_{1}+b_{2}x_{2}+...+b_{m}x_{m}+b_{0}$$

with *ŷ* for the predicted property (dependent variable), and *x*_1_, *x*_2_, ..., *x*_*m*_ for *m* descriptors (independent variables). The model parameters are the regression coefficients, *b*_1_, *b*_2_, ..., *b*_*m*_, and the intercept, *b*_0_ (which is zero for mean-centered data); they can be estimated by various regression methods using an appropriate calibration data set.

The principal aims of model building are small prediction errors ∣*y* - *ŷ*∣ for data from substances in a test set that have not been used during model creation. For many QSPR/QSAR problems a small set of well understood, relevant descriptors is not known, and therefore a large set of descriptors is tried by applying appropriate mathematical variable selection and modeling methods. It is essential for a good model to have an appropriate (optimized) model complexity to avoid underfitting (a too simple model), and overfitting (the model is too much adapted to the calibration data) [3]. These aims are mostly achieved by cross validation techniques (as in this contribution, see below) or bootstrap techniques.

This review - partly in tutorial style - gives a user-oriented description of how to make linear QSPR/QSAR models by PLS, a well-proven method of multivariate data analysis (chemometrics), and using free, standard software. Recently, a comprehensive overview about machine learning methods for property prediction in chemoinformatics has been reported [4]. Here, the emphasis is on (1) a comparison of some basic variable selection methods, and (2) a strategy for evaluating variable selection methods in terms of the performance for test set objects. The presented demonstration example is about the modeling of the gas chromatographic retention index (*y*) of a set of 209 polycyclic aromatic compounds (PACs) [5]. Approximate 3D structures have been created by *Corina* software [6], and molecular descriptors have been computed by *Dragon* software [7]. All other used software is written in *R* [8]. The programming environment *R* is freely available, and a goal of the contribution is to promote the use of this rapidly developing tool in QSPR/QSAR. A complete set of *R* functions (including import of *Dragon* result files) and data sets as used in the example are provided as free supplementary material [9].

We start with introductory remarks about the *R* programming and software environment. Next, a short, user-dedicated introduction into the widely used PLS regression method is given. The basics of the evaluation of regression models are summarized. The strategy “repeated double cross validation” (rdCV, [10]) is explained as a powerful approach for optimizing the complexity of PLS regression models, together with an independent cautious estimation of the model performance for new cases. Then, basic, strictly defined variable selection methods are briefly described. An example includes the application of these variable selection methods, followed by a careful examination of the resulting variable subsets by rdCV. Finally, a QSPR model with 22 descriptors is presented.

## Methods

### Software environment R

*R* [8] is an open source software environment originally devoted to statistical computing. The structure of *R* programs has similarities with the commercial product *Matlab* [11] and the free software *Octave* [12], both widely used for numerical computations. Many people appreciate *R* because it is widely distributed and rapidly developed, independent from any company or a single user group. *R* was basically developed by Ross Ihaka and Robert Gentleman in 1994 [13]. More than 4000 contributed packages are available now (making it sometimes troublesome to find appropriate ones [14]). Latest implementations are not only for statistics but also for applications in mathematics, chemistry, biochemistry (e. g., *Bioconductor* [15], providing tools for the analysis of genomic data), medicine, geology, and finance (e. g., the non-profit *Rmetrics* Association, https://www.rmetrics.org).

*R* is freely available under the GNU General Public License. It can be easily installed from http://cran.r-project.org for the operating systems Windows, Linus or Mac. Like similar software products, *R* requires some experience in using computer programs; in its standard version it is command controlled but not mouse controlled. However, a number of user-oriented functions allow the application of high-level numerical and other formal methods without special skills.

### PLS regression

PLS stands for partial least-squares, and is a linear, multiple regression method (several *x*-variables, one or several *y*-variables) frequently used in chemometrics for calibration models [3, 16, 17]. PLS (like similar methods) has advantages that are especially useful - even necessary - for typical data sets in chemometrics (including QSPR/QSAR): (1) Data with highly correlating *x*-variables can be used (correlating *x*-variables are even considered as useful “duplicate” measurements). (2) Data sets with more variables than samples can be used. (3) The complexity of the model can be controlled by the number of components, and thus overfitting can be avoided and maximum prediction performance for test set data can be approached. (4) Several software packages are available. In this review only a few features of PLS - essential for the user - are outlined; for technical details see [3, 17–20]. Note that for PLS different mathematical approaches have been published and implemented into the software, so it is less strictly defined than the traditional method OLS (ordinary least-squares regression, mostly not directly applicable to chemistry-related data because of correlating variables and a larger number of variables than number of objects), and PCR (principal component regression, similar to PLS).

In principle, regression can be carried out directly with the variables (e. g., by OLS) but in the powerful methods PLS and PCR is performed via a small set of intermediate linear latent variables (the components). A component is defined by ***t*** = ***X p***. Where *X* is the mean centered variable matrix (*n* × *m*) for *n* objects and *m* variables; ***p*** is a loading vector defining an appropriate direction in the *m*-dimensional variable space; ***t*** is a vector with the *n* values of the component (the scores). According to the concept of PLS, ***p*** defines a direction which gives the maximum covariance between the modeled property and the scores. The empirical covariance between two mean-centered variables (here the vectors ***y*** and ***t***) can be calculated by ***y***^**T**^ ***t*** / (*n*-1); covariance considers the correlation between the variables and also their variances (therefore, it depends on the scaling of the variables considered - in contrast to the Pearson correlation coefficient, which is the basis of OLS). The concept of PCR for defining the components is slightly different, as a loading vector is determined that gives scores with the highest variance (highest spread in x-space), thus ignoring ***y***. Further PLS- or PCA-components can be calculated by repeating the strategy. While for PCA (principal component analysis) the concept is clearly defined (orthogonal loading vectors and uncorrelated score vectors), different strategies can be applied for PLS (often the condition uncorrelated scores and maximum covariance between scores and ***y***). Typically, 1 to 15 scores are used as independent variables in OLS regression. The number of components, *A*, determines the complexity of the model and has to be optimized. If *A* is too small the model is too simple (underfitted) and gives high errors in the prediction of *y*. If *A* is too large the model is highly fitted to the used calibration data, but not optimally generalized for new data; therefore, calibration data give very low errors, however, new data produce high errors (overfitting). It is a great advantage of PLS and PCR to provide this optimization (although it requires an appropriate strategy and computational effort, see below). Because PLS considers *y*, the optimum *A* is often smaller than for PCR and therefore better fulfills the concept of parsimony for models (however, PLS and PCA often yield similar model performances).

The mathematics is usually not of primary interest for the user and is mostly hidden in the software. Essential is the structure of the resulting regression model: it is the same for PLS, PCR, and many other methods, as defined in Equation (1). Regardless of how many components have been found to be optimal, and the strategy applied, we have a regression coefficient, *b*_*j*_, for each variable *j* (and an intercept). In general, the resulting regression coefficients are different for PLS and PCR, and also the prediction performances of the models are somewhat different.

Software for PLS is contained in the *pls* [20] and *chemometrics* [3, 21, 22] *R*-packages. *R* functions easier to use for routine applications of PLS and model evaluation are included in the supplementary material.

### Evaluation of regression models - overview

Any prediction model makes sense only if appropriate performance criteria are known. In the case of calibration models (modeling/predicting a continuous property *y*) the residuals (prediction errors), *e*_*i*_

$$e_{i}=y_{i}-{\hat{y}}_{i}$$

are the basis for most performance measure, with *y*_*i*_ for the given (experimental, “true”) value and *ŷ*_*i*_ the predicted (modeled) value for a property of an object *i*. The great number of differently defined performance measures and also the various strategies for selecting the objects used to calculate prediction errors may be confusing to some users. We focus here on elementary concepts as follows.

Because in chemometrics and QSPR/QSAR the number of objects is often rather small, adequate strategies have to be applied for splitting the data into a calibration set (used for making a model (including optimization of the complexity, say the number of PLS components), and a test set (for estimation of the model performance). Final performance measures must be from test set data, and no (further) model optimization must be done from test set results. Performance data obtained from the calibration set (during optimization) are only of secondary interest. Also note that a performance measure is an estimated quantity, which has some variation depending on the (usually random) split into calibration and test set. Therefore, it is better to repeat the process and exhibit distributions (represented, e g., by boxplots) for performance criteria rather than single numbers (from a single random split).

Suppose we have *z* prediction errors, *e*_*i*_ (in the simplest case, *z* is the number of objects in a single test set). If *z* is not very small (say >20) a graphical representation of the distribution of *e*_*i*_ gives an informative picture of the prediction errors to be expected. Often this distribution is similar to a normal distribution with a mean near zero, and then a single parameter, the standard deviation of *e*_*i*_, is a good and widely used measure for the model performance, known as standard error of prediction (SEP). SEP is given in units of the property *y*. In case of a normal distribution of the prediction errors, about 95% of them are within the tolerance interval ±2SEP. Note that SEP refers to test set data and is therefore the relevant measure for future uses of a final model. Equivalent measures derived from prediction errors obtained during cross validation with the calibration set, must be clearly marked as such. Sometimes SEC, standard error of calibration, is used if a model is applied to the calibration data from which the model has been developed; SEC maybe too optimistic for new cases. Many other error criteria appear in literature, such as MSE (mean squared error = arithmetic mean of squared errors), RMSE (root mean squared error = square root of MSE), PRESS (predicted residual error sum of squares = *z* MSE).

An evident performance measure is the squared correlation coefficient, *R*^2^, (usually Pearson, rarely the robust measures Spearman or Kendall) between *y* and *ŷ* - preferably accompanied by the corresponding scatter plot. Again, it is essential that the *ŷ* -values come from data of the test set.

Mathematical variable selection is often necessary in QSPR/QSAR because of a large number of available descriptors - and the usual lack of understanding the relationships between the descriptors and the property. When comparing the performances of models with a different number of variables, it has to be considered that *R*^2^ becomes larger as the number of variables increases. Therefore, different criteria are used that apply a penalty to a larger number of variables, e. g., the adjusted squared correlation coefficient

R2ADJ=1-(n-1)(1-R2)/(n-m-1)

with *R*^2^ for the Pearson correlation coefficient, *n* the number of objects, and *m* the number of variables. For the stepwise variable selection described below, we use the Bayes information criterion

BIC=nlog(RSS/n)+mlog(n)

with RSS for the sum of the squared residuals *e*_*i*_ (*i* = 1 ... *z*), and log for the logarithm with base *e*. A similar criterion is AIC, Akaike‘s information criterion,

AIC=nlog(RSS/n)+2m

which has a smaller penalty for large *m* (for *n*>7). In terms of parsimony, a model with a smaller BIC (or AIC) is preferable, however, the values for BIC or AIC are meaningless. These three criteria require *n* > *m* as is the case for OLS which is often applied during variable selection, e. g., in stepwise selection. Furthermore, they are usually calculated for the calibration set in order to keep the computational effort reasonable. However, that means, that a variable set giving, e. g., a lower BIC than another one is not necessarily better in terms of SEP for test set objects. The strategy rdCV, described below, is capable to compare model performances carefully for given variable sets.

### Repeated double cross validation (rdCV)

First the standard procedure for cross validation (CV) is briefly explained, and then rdCV is described, a strategy for independent estimations of the optimum model complexity and the model performance.

**Figure 1:**
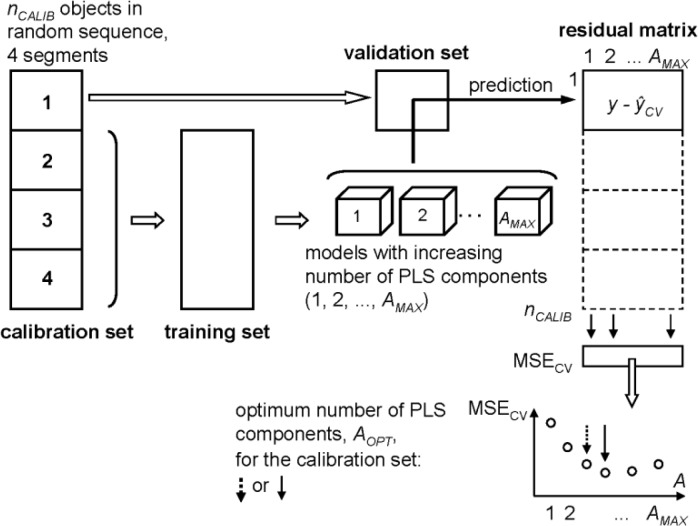
Cross validation (CV) for an estimation of the optimum number of PLS components, *A*_*OPT*_, from a calibration set, using 4 segments [3]. *A*_*OPT*_ can result from the global minimum of MSE_CV_ (mean of squared prediction errors in CV) or e. g., by applying the one standard error rule (dashed arrow) for a more parsimonious model

Typical data sets in QSPR/QSAR contain only a rather small number of objects (say *n* from 30 to 500). For the split into a calibration set and a test set, the so-called resampling methods are applied, e. g., CV or bootstrap techniques. The typical CV strategy is described here as often used to estimate the optimum number of PLS components (*A*_*OPT*_) from a calibration set (with *n*_*CALIB*_ objects), see Figure 1. The *n*_*CALIB*_ objects are randomly split into *s* segments (parts) of approximately equal size, with the number of segments between 2 and *n*, often between 3 and 7. One segment is left out as a validation set. The other *s*-1 segments are used as a training set to create models with increasing complexity, e. g., models with 1, 2, 3, …, *A*_*MAX*_ PLS components (e. g., *A*_*MAX*_ = 10). The models are separately applied to the objects of the validation set resulting in predicted values of *y* for the different model complexities. This procedure is repeated so that each segment is a validation set once. The result of this CV is a matrix with *n*_*CALIB*_ rows and *A*_*MAX*_ columns containing the predicted values *ŷ*_*CV*_ for all objects of the calibration set and all considered model complexities. From this matrix and the given target values of *y* a residual matrix (matrix with prediction errors) is computed, and the MSE_CV_ (mean of squared errors) is calculated for each model complexity. For *A*_*OPT*_ the number of PLS components with the smallest MSE_CV_ can be chosen, but usually a somewhat lower value is taken (to avoid overfitting) by applying a heuristic algorithm, like e. g., the one standard error rule [3, 19].

The prediction errors obtained in CV should not be used for an estimation of the model performance for new cases because they are usually too optimistic. Therefore, besides the calibration set a test set with objects not used in the estimation of *A*_*OPT*_ is necessary. For this purpose the total number of objects (*n*) is randomly split into a calibration and a test set - again by CV (thus giving a double CV strategy). The outer CV loop makes the split into calibration and test set, the inner CV loop is used to estimate *A*_*OPT*_. Test set predictions (*ŷ*) are obtained by making a model from the whole actual calibration set with *A*_*OPT*_ PLS components as estimated from this calibration set. After a complete double CV run we have a test set predicted *ŷ* for each object from which a SEP can be calculated.

The SEP obtained is a single estimation and depends on the applied (random) splits of the objects into segments. Only considering a single estimation of SEP can be very misleading, especially when comparing different models. An appropriate non random split of the objects into “highly representative” sets, e. g., by sampling techniques, is usually not applicable for multivariate data. However, the variability of SEP (and other measures) can be estimated by repeating the double CV with different random splits (typically with *r* = 20 to 100 repetitions). This repeated double cross validation (rdCV) [10] yields *r* estimations of SEP that can preferably be represented by a boxplot. In this way different models (e. g., from different variable sets) can be compared considering the variability of the used performance measure. The rdCV strategy also demonstrates the variability of *A*_*OPT*_; the number of estimations for *A*_*OPT*_ being *r* times the number of segments in the outer CV loop. A final value, *A*_*FINAL*_, may be the value of *A*_*OPT*_ with the highest frequency. Alternatively a set of several values for *A*_*FINAL*_ may be considered resulting in several models and combining the predictions by averaging or a consensus strategy.

A final model is computed from all *n* available objects using *A*_*FINAL*_ PLS components - of course without any further model optimization. The SEP for *A*_*FINAL*_ PLS components as obtained during rdCV is a good estimation of the model performance for new cases.

The whole rdCV strategy - up to a final model - can be automatically applied by the provided *R* software that optionally also produces a number of diagnostic plots.

### Variable selection

Data sets in QSPR/QSAR typically have some hundred to some thousand descriptors (*x*-variables), hence a (drastic) variable selection appears useful or even necessary. PLS and similar regression methods can use data sets with more variables than objects and also highly correlating variables; nevertheless there are arguments for variable selection: (1) Use of many variables gives a better fit of the model for the training data. However, we are usually not primarily interested in this effect but in an optimum prediction performance of the test data. Therefore, a reduction of the variables can avoid overfitting and lead to an improved prediction performance. (2) A model with many variables is practically impossible to interpret. An interpretation seems feasible only if no more than about a dozen variables are used in the model.

An exhaustive search for the best subset of variables is not possible for data sets with more than 20 to 30 variables, and therefore in practice all results from variable selection are suboptimal. An optimum variable selection for *m* original variables would require the test of 2^*m*^ -1 variable sets - this number is about a million for *m* = 20; ideally with a strategy that optimizes the performance for test set objects (rdCV or double bootstrap). This is computationally not feasible - not even for only 20 variables. Therefore, approximate fast methods are applied and usually the fit of the training data is used as performance measure rather than e. g., SEP for test data. Another hardly solvable aspect for practical problems is whether variable selection should be performed with subsets (by CV) of the objects (probably resulting in several different “optimum” variable subsets) or with all objects (with the danger of too optimistic results). Note, that merging of two good variable subsets will not necessarily result in an improved performance - it may even become worse than the performance of each subset.

Considering the unsolvable problems of variables selection on one hand, and the need for variable selection on the other hand, the following strategy is claimed here: Variable selection is performed with all *n* available objects thus exploiting all information present in the available data (the most evident approach). Several approaches for variable selection are applied in parallel and the controlling parameters within the methods are varied. Result may be 5 to 20 variable subsets (of different size and contents). No performance measures obtained during variable selection are considered for the performance of final models. Consider the resulting variable subsets as suggestions by a good, experienced (but not error-free) friend - or maybe coming from a *deus ex machina* (also the original variable set is something like this). These variable subsets (and also the complete set) are then carefully tested for their capability to produce models with a high performance for test set objects - in this work by rdCV. Typically, each variable set gives a set of (say *r* = 20 to 100, number of repetitions in rdCV) estimations of SEP. The power of the different variable sets can be compared visually from the boxplots of *r* SEP values. Alternatively statistical tests can be applied (e. g., t-test or U-test for the comparison of central values) or Kolmogorov-Smirnov test for the comparison of distributions.

The methods for data preprocessing and variable selection as used in the example, discussed below, are now briefly described (functions in *R* provided).

In a simple cleaning step the constant or “almost constant” variables have been eliminated. “Almost constant” means that a variable has the same value in all but a maximum of *k* objects. For the example *k* = 3 was used. After cleaning, a single variable selection method or a combination of them has been applied. The four variable selection methods applied here are as follows.

1. Select variables with a very high correlation to *y* (modeled property). For the example, sets with 5 to 200 variables possessing highest squared Pearson correlation coefficients with *y* have been selected.
2. Delete variables with a very high correlation to another variable. The squared correlation coefficient, *R*^*2*^(*x*_*g*_, *x*_*h*_) between all pairs of variables (*g*, *h*) is checked. If it is higher than a given limit one of the variables is deleted. The deleted variable has the higher sum of squared correlation coefficients to all other variables. For the example, limits between 0.9999 and 0.9 for *R*_*XX*_^2^ have been tested.
3. Select variables with high absolute standardized regression coefficients in a PLS regression model. This method is intended to reduce noise and to give a generalization, resulting in a better performance for new cases. For this purpose, an optimized PLS regression model has been created from autoscaled data (each variable is scaled to a mean of zero, and a variance of one) by the rdCV strategy. A set of variables is selected possessing highest ∣*b*_*j*_∣, *j* = 1... *m*, see Equation (1) - based on the assumption that these variables contribute most to the model. For the example, rdCV was applied with 3 segments in the outer loop (test set split), 5 segments in the inner loop (estimation of *A*_*OPT*_), and all *n* = 207 objects were used with 50 repetitions. The final optimum number of PLS components, *A*_*FINAL*_, was the *A*_*OPT*_ value (among 3×50 estimations) with highest frequency. A PLS model with *A*_*FINAL*_ components was then made from all *n* objects, and the resulting regression coefficients were considered for variable selection. Sets with 20 to 300 variables possessing largest ∣*b*_*j*_∣ have been tested.
4. A stepwise selection of variables, using the BIC criterion (Equation (4), has been used. A “forward” stepwise selection procedure starts with an “empty model” where the dependent variable *y* is explained only by the intercept, and adds in each step one variable until no further improvement is possible. A “backward” stepwise selection procedure starts with the “full model” using all explanatory variables, and removes in each step one variable until no further improvement is possible. One can also go in “both” directions by adding or removing one variable at a time, starting either from the empty or from the full model. Since in the example (see below) we have more variables than observations, we cannot start with the full model. Here we use two strategies: forward stepwise selection, and stepwise selection in both directions (forward/backward). The algorithm of a newly developed *R* function applies one of these strategies and stops until no more improvement can be done or until a certain number of steps or a pre-defined computing time is reached. Although the new function is much faster - especially for more than about 100 variables - than the long existing *R* function *step()*, about one hour computation time is necessary for a data set with *n* =207 and *m* = 2688 (resulting e. g., in 22 selected variables).

We only mention other powerful variable selection methods, not used in this mini review article: Lasso regression [3, 23], genetic algorithms [24], and heuristic replacement methods [25].

## Example

### Data

**Figure 2:**
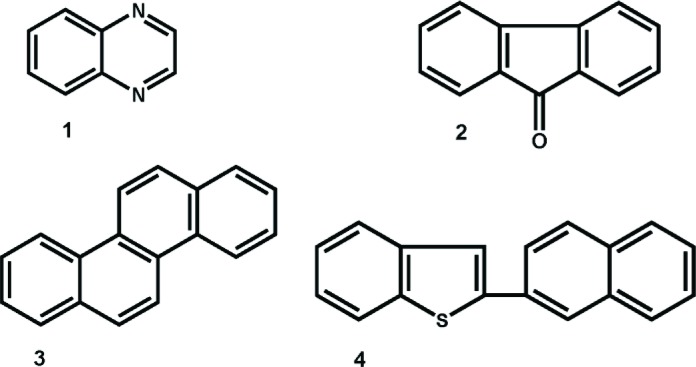
Examples of polycyclic aromatic compounds (PACs) used and their GC retention indices, *y* [5]. **1**, quinoxaline, *y* = 220.37; **2**, 9-fluoranthene, *y* = 294.79; **3**, chrysene, *y* = 400.00 (reference); **4**, 2-(2'-naphthyl)-benzene[*b*]thiophene, *y* = 428.11.

For a set of 209 polycyclic aromatic compounds (PACs) the 2D chemical structures have been drawn manually by a structure editor software and stored in an SDF-file in Molfile format [26]. Recently, the *R* package *RMol* [27] has been released. It is available from http://sourceforge.net/p/rmol-toolset, and contains functions for handling chemical structure data in Molfile format and provides connections to graph theory software, e. g., *QuACN* [28]. The 209 PACs have molecular formulae with atom ranges C_8-24_ H_6-24_ N_0-2_ O_0-2_ S_0-2_; examples with retention indices (see below) are shown in Figure 2.

Approximate 3D-structures with all H-atoms explicitly given, have been created from the 2D structures by *Corina* software [6, 29, 30]. For the calculation of molecular descriptors either an SDF-file with these 3D-structures including all H-atoms, or an SDF-file with 2D atom coordinates and no H-atoms (H-depleted graphs) have been used [31].

*Dragon* software, version 6.0 [7], has been used to compute molecular descriptors [1]. From the 3D structures with all H-atoms explicitly given, a set of 2772 descriptors has been generated (excluding constant descriptors). From the 2D H-depleted structures a set of 1620 descriptors has been generated (excluding constant descriptors). Output from *Dragon* consists of three files in text format (one with descriptor values, one with descriptor names, and one with object/structure names). These files have been imported into the *R* environment yielding matrices (209×2772) and (209×1620).

The *y*-variable to be modeled is the gas-chromatographic (GC) retention index, published by Lee et al. [5]. This index is based on the reference values 200, 300, 400, and 500 for the compounds naphthalene, phenanthrene, chrysene, and picene, respectively, thus the reference indices are given by the number of (condensed) rings in these structures times 100. Values of *y* are between 197.0 and 503.9, mean is 338.1, and standard deviation is 80.8. Aim of QSPR work is a model that predicts *y* from the *x*-variables (descriptors) with small errors, and preferably using only a subset of the variables. The performance of the model has to be estimated for new cases (PACs that are not contained in the currently available data set). Multivariate regression models for this GC retention index for the same set of compounds but using only a subset of the descriptors applied here, have been reported [3, 10], as well as models with other descriptors [32, 33].

### Results

**(A)** The only data cleaning was elimination of constant and almost constant variables. A variable was considered as “almost constant” if all structures have the same value except three structures or less (variables were rounded to 6 decimals). From the 1620 descriptors obtained from 2D structures without H-atoms 53 were deleted, resulting in a matrix ***X***_***2D***_ (209×1567); from the 2772 descriptors from 3D structures with all H-atoms 84 were deleted, resulting in a matrix ***X***_***3DH***_ (209×2688). These two matrices were the starting data in all variable selection procedures. For the judgment of variable selection methods the rdCV strategy has also been applied to the data set with all variables.

**Figure 3:**
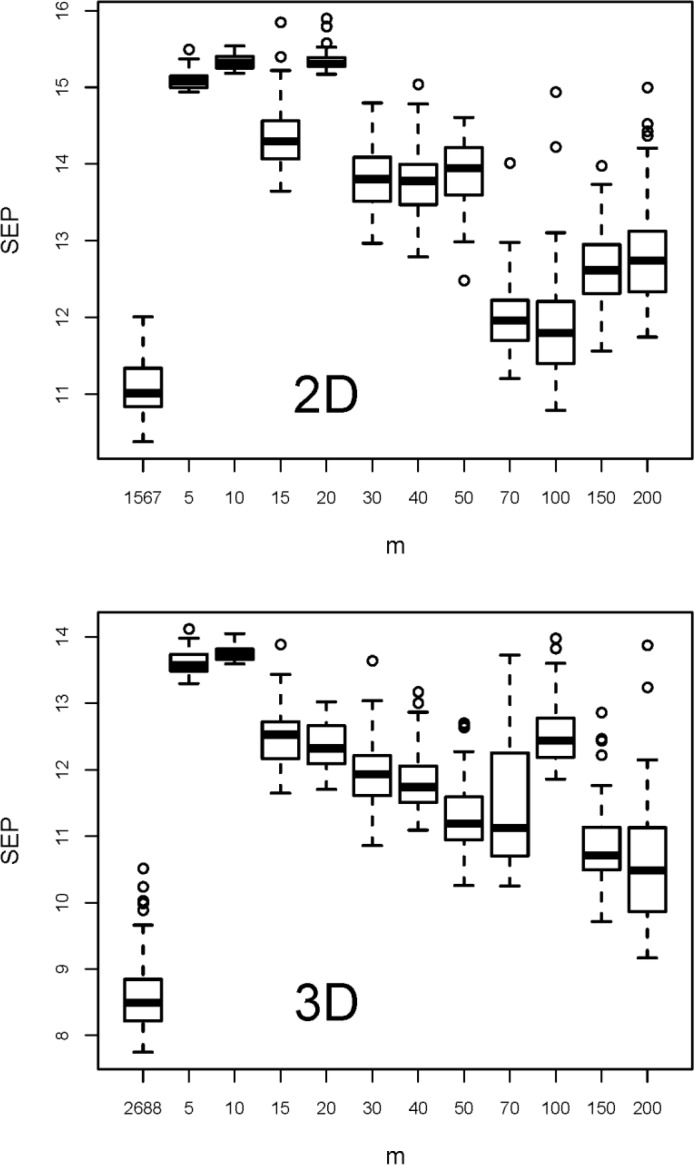
Results from variable selection by maximum squared Pearson correlation coefficient with *y*. Boxplots denote 50 estimations of SEP obtained by rdCV; *m* is the number of selected variables; for comparison results for the full variable sets (*m* = 1567 for 2D descriptors, and *m* = 2688 for 3D descriptors) are shown.

**(B)** The first variable selection method tested was selection of variables with highest squared Pearson correlation coefficient with property *y*. This evident and often proposed approach has been applied to both X-matrices with the aim also to check whether the 3D descriptors are of benefit for the model performance or not. Eleven subsets with 5 to 200 variables have been selected from each X-matrix. These subsets and the original variable sets have been tested by rdCV, and the summarized results are shown in Figure 3. The 50 repetitions applied in rdCV give 50 estimations for SEP (each from *n* = 209 test set objects) for each variable set. A boxplot visualizes the center value and the variation of the 50 estimations. Note that in a boxplot [3, 34] the thick horizontal line denotes the median (a robust measure for the central value). The lower and upper borders of a box are the first and third quartiles, *Q*_1_ and *Q*_3_, respectively. The lower whisker extends to the smallest value in the range *Q*_1_ to *Q*_1_ - 1.5 IQR, the upper whisker to the largest value in the range *Q*_3_ to *Q*_3_ + 1.5 IQR, with IQR being the interquartile range *Q*_3_ - *Q*_1_, a robust measure for the spread. Outliers are plotted as individual points, and the width of the box has usually no meaning.

For both variable sets models using all variables show smaller prediction errors than the variable subsets obtained by this selection method. Therefore, we conclude that - at least for the investigated data sets - a univariate variable selection by considering maximum correlation with *y* is not successful. This effect has also been found with other data sets, e. g., in [35].

Furthermore we see that 3D descriptors improve the model performance considerably; the medians of SEP for 2D and 3D being 11.0 and 8.5, respectively, without an overlap of the distributions. Because in all other variable selection methods tested here, 3D descriptors gave better results than 2D descriptors (results not shown), in the further discussions only the 3D descriptors are considered. The benefit of 3D descriptors is treated here on a purely empirical basis, only considering the performance of QSPR regression models; a recent study [36] discusses fundamental problems with 3D descriptors, e. g., their temperature dependence.

**Figure 4:**
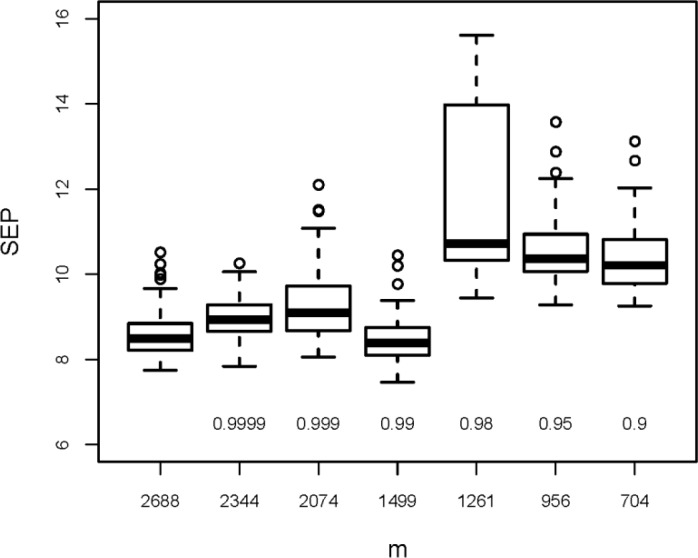
Results from elimination of variables with a high correlation to another *x*-variable (limits applied for the squared Pearson correlation coefficient, *R*_*XX*_^2^, are between 0.9999 and 0.9). Boxplots denote 50 estimations of SEP obtained by rdCV; *m* is the number of selected variables; for comparison, results for the full variable set (*m* = 2688) are included.

**(C)** Elimination of *x*-variables with a high squared Pearson correlation coefficient (*R*_*XX*_^2^) to any other *x*-variable was tested only for ***X***_*3DH*_ (209×2688). The values of *R*_*XX*_^2^ for the 3611328 pairs of *x*-variables are mostly low (median is 0.128), and only 1% are > 0.955. The limits applied for *R*_*XX*_^2^ were 0.9999, 0.999, 0.99, 0.98, 0.95, and 0.9. If two variables have a higher *R*_*XX*_^2^ than the limit, one of them is eliminated (see above in section “Variable selection”). Figure 4 shows the results (again boxplots for 50 repetitions in rdCV, together with the results obtained from all 2688 variables). Elimination of very highly correlating variables, that means elimination of identical or almost identical variables (limit for *R*_*XX*_^2^ between 0.9999 and 0.99), does not influence the model performance, but elimination of variables with less correlation (*R*_*XX*_^2^ between 0.98 and 0.9) worsens the model performance considerably. Note that a single random split into a calibration and a test set (instead of 50 repetitions) may give misleading results because the - in some cases very high - variability of SEP would not be considered. First eliminating highly correlating variables (*R*_*XX*_^2^ > 0.9999) and then selecting 5 to 100 variables with highest correlation with *y* produced even higher prediction errors than only using the latter method (results not shown).

**Figure 5:**
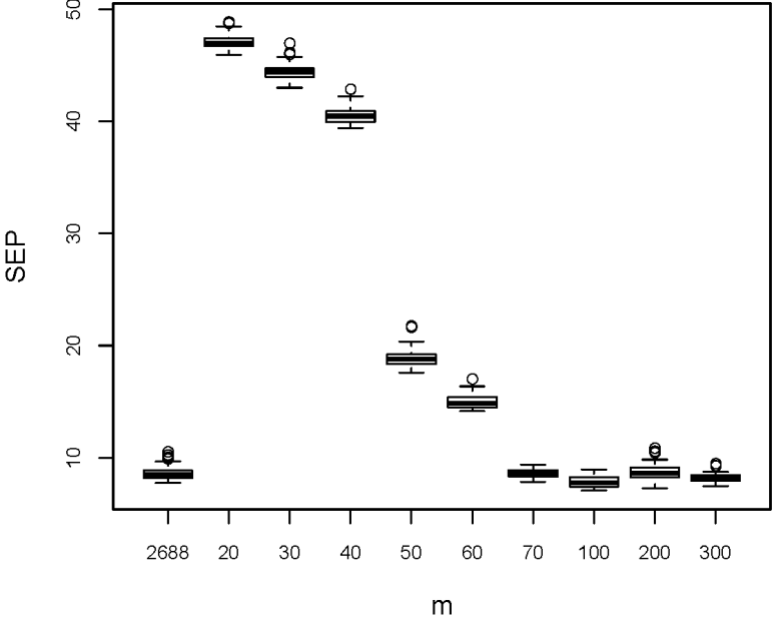
Results from variable selection considering maximum absolute standardized regression coefficients of a PLS model (obtained via rdCV from all *n* = 209 objects and all *m* = 2688 variables). Boxplots are constructed from 50 estimations of SEP obtained by rdCV; *m* is the number of selected variables; for comparison, results for the full variable set (*m* = 2688) are included.

**(D)** A multivariate approach for variable selection uses the standardized regression coefficients of a multiple regression model. A PLS model has been calculated from the complete autoscaled descriptor set ***X***_*3DH*_ (209×2688), with the number of PLS components optimized by rdCV (*A*_*FINAL*_ = 6 with SEP = 7.3; SEC = 5.0). For variable selection, sets with 20 to 300 variables possessing maximum absolute regression coefficients have been selected, and then tested by rdCV as described above. Figure 5 shows the results - as before boxplots for 50 repetitions in rdCV, together with the results obtained from all 2688 variables. We see, that at least 70 variables are necessary - selected by this method - for achieving the same performance as with all variables. A slight improvement is obtained for a set of 100 variables, obviously constituting a good compromise between underfitting and reduction of noise. Because the separation of the boxplots for 2688 and 100 variables cannot be seen in the Figure, three characteristic numbers (first quartile, median, and third quartile of the 50 SEP estimations) are given: for *m* = 2688 the values are 8.2, 8.5, and 8.8; for *m* = 100, the values are 7.4, 7.8, and 8.2, respectively.

**Figure 6:**
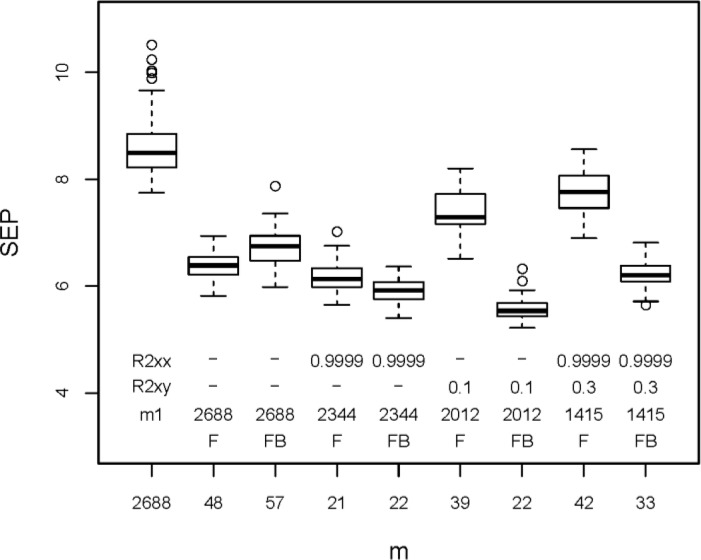
Results from stepwise variable selection. Boxplots are constructed from 50 estimations of SEP obtained by rdCV. For comparison, results for the full variable set (*m* = 2688) are included. The stepwise strategies F (forward) and FB (forward/backward) selected from the 2688 variables subsets with 48 and 57 variables, respectively, showing a considerable reduction of SEP. Pre-filtering before stepwise selection reduced the number of selected variables to 21 to 42, and in some cases improved the model performance (selected examples shown). R2xx is the applied *R*_*XX*_^2^ for eliminating *x*-variables with a very high correlation to another *x*-variable; R2xy is the applied *R*_*XY*_^2^ for eliminating *x*-variables with a very low correlation to *y;* m1 is the number of variables after pre-filtering. Models with lowest SEP values have been calculated from 22 variables obtained by pre-filtering with *R*_*XY*_^2^ < 0.1, and then forward/backward stepwise variable selection.

**(E)** Stepwise variable selection has been performed with the forward (F) and the forward/backward (FB) strategy applied to the complete descriptor set ***X***_*3DH*_ (209×2688). Furthermore, pre-filters have been used before stepwise selection: (a) elimination of *x*-variables with a squared Pearson correlation coefficient to another *x*-variable, *R*_*XX*_^2^ > 0.9999; (b) elimination of *x*-variables with a squared Pearson correlation coefficient to *y*, *R*_*XY*_^2^, below a limit (varied between 0.1 and 0.5); (c) a combination of both. This pre-filtering excludes highly correlating variables and variables with very low correlation to *y*. Figure 6 shows selected results - again boxplots for SEP from 50 repetitions in rdCV. Stepwise variable selection with all 2688 variables resulted in much smaller sets with 48 (F) and 57 (FB) variables, respectively, showing a considerably improved performance. Pre-filtering achieved in some cases a further, small improvement of the performance and always reduced the number of selected variables (compared with no pre-filtering). Best models have been obtained with first an elimination of variables with *R*_*XY*_^2^ < 0.1, and then forward/backward selection, resulting in 22 selected variables (median of 50 SEP values from rdCV is 5.9, compared to 8.5 for models with all variables).

**(F)** The results from the used variable selection methods are summarized as follows.

Selection of variables with high correlations to *y* produced worse models than using all variables. Elimination of highly correlating variables gave similar or worse models than using all variables. Selection of variables with highest absolute standardized regression coefficients (in an optimized PLS model from all variables) had no or only small positive influence on the model performance. Stepwise selection gave variable subsets with highest performances for the prediction. A previous elimination of *x*-variables with a very low correlation to *y* (*R*_*XY*_^2^ < 0.1) slightly improved the models from variable sets obtained by stepwise selection.

**Figure 7:**
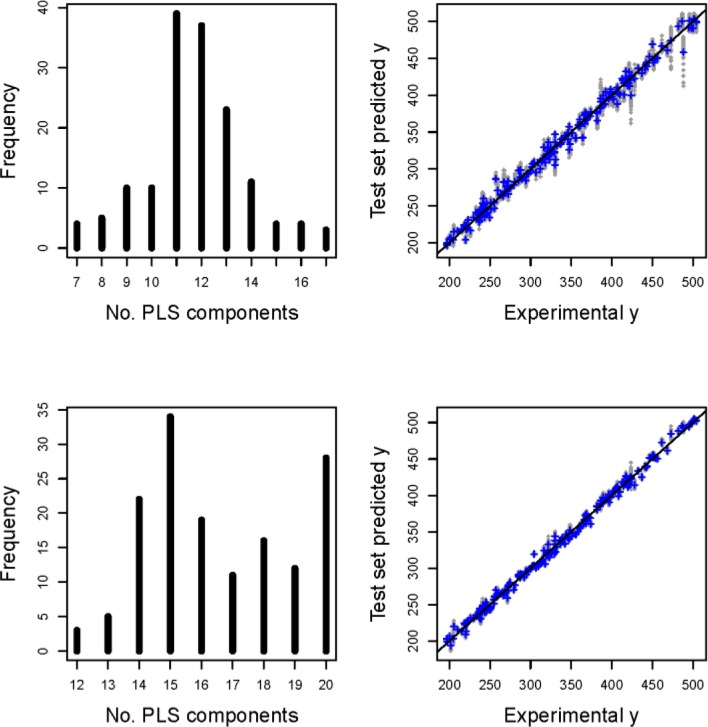
Diagnostic plots from rdCV. Upper part is for *m* = 2688 variables, lower part for *m* = 22 variables selected by first eliminating variables with *R*_*XY*_^*2*^ < 0.1 (squared Pearson correlation coefficient between *x*-variable and *y*), and then stepwise selection forward/backward - this is the variable set with best prediction performance. At the left hand side are histograms for the estimation of the optimum number of PLS components (150 estimations each); the value with maximum frequency has been taken as *A*_*FINAL*_. At the right hand side the test set predicted *y*'s are plotted versus the target values (experimental *y*). In gray are the results from the 50 repetitions in rdCV (for *m* = 22 very close together and therefore mostly hidden), in blue are the means of 50 predictions.

**(G)** A final model for future use has been derived as follows: From the many suggestions for variable selection a subset with 22 descriptors (out of 2688, including some 3D descriptors, see Figure 6) was most promising because the test set predictions - as obtained from rdCV - have smallest errors with a mean for SEP of 5.6. For approximately normally distributed errors (which is the case in this example) a prediction error of ±2SEP, equal to about ±12, can be given (95% tolerance interval) for predicted *y*'s of new objects. rdCV also gives a final estimation for the optimum number of PLS components for these 22 variables, namely *A*_*FINAL*_ = 15. Note that this relative high number for *A*_*FINAL*_ is typical for a successful variable selection. Figure 7 shows diagnostic plots from rdCV for this small, optimum variable set and for all 2688 variables.

A final model has been calculated from all *n* = 209 objects, using these 22 selected variables and *A*_*FINAL*_ = 15 PLS components. No further optimization of model parameters is advisable at this stage, and the SEC of 4.8 is only a plausibility measure; it is - as expected - smaller that SEP (ca 5.6) obtained from test set objects in rdCV. Note, SEP is relevant for future use of the model.

The regression coefficients (*b*_1_, ..., *b*_22_) of the final model with the 22 selected variables (denoted by the descriptor codes as used in *Dragon* [7]) are listed here, rounded to 3 decimals: “nCsp2” (24.198), “Rperim” (3.629), “D/Dtr12” (0.074), “DECC” (7.333), “AAC” (58.633), “SpPosA_Dt” (3.919), “VE2_B(p)” (-6.714), “ATSC4p” (2.562), “P_VSA_i_2” (-2.678), “SpMAD_AEA(bo)” (-0.079), “SM11_AEA(bo)” (10.583), “Eig11_AEA(dm)” (-5.52), “Ho_G/D” (-1.842), “RDF060m” (-1.216), “RDF010s” (11.094), “Mor05v” (-1.234), “Mor02e” (1.016), “Mor10p” (4.298), “Mor24s” (3.97), “E1m” (6.367), “H3m” (33.932), “H5s” (9.297). Intercept (*b*_0_) is 338.086. Note that these regression coefficients are not standardized, but refer to the original descriptor values. Therefore, their (absolute) values cannot be interpreted in terms of importance for the model. About half of the descriptors are from 3D structures with H-atoms. Except the first one (“nCsp2”, number of sp2 hybridized carbon atoms, all others are based on concepts from graph theory and other mathematics, probably difficult to interpret by most chemists. A discussion of the definition and possible chemical background of the descriptors is also out of the scope of this contribution, see [1]. This situation is typical for QSPR/QSAR applications; the models are “black” or “gray” and cannot be sufficiently interpreted. Therefore, a careful evaluation of the model performance is an absolute requirement, as well as an estimation of the applicability domain (not discussed here) for further use of a model. Because of the not sufficiently known relationships between chemical structures (their representation by molecular descriptors) and properties or activities such empirical regression models are often accepted in QSPR/QSAR.

**(H)** Here are examples of *R*-functions for the described variable selection methods and other tasks [9]. X is the variable matrix (*n* × *m*), y is the vector with the properties (*n* values). Result of variable selection is always a logical vector (sel) with *m* values TRUE or FALSE that define the selected variables; sel is preferably stored as RData file. e. g., by save (sel, file = “sel.RData”). *R* packages used are e. g., *chemometrics* [21] and *pls* [20].

**sel <- varsel_almost_const (X, k = 3)**

Delete constant or almost constant (parameter *k*) variables.

**sel <- varsel_corr_xy (X, y, m_sel = 10, r2_xy_limit = 0.7)**

Select a maximum of m_sel variables with a squared Pearson correlation coefficient with *y* > r2_xy_limit.

**sel <- varsel_corr_xx (X, r2_xx_limit = 0.9)**

Delete all variables with a squared Pearson correlation coefficient with another *x*-variable > r2_xx_limit.

**sel <- varsel_pls_regr_coeff (X, y, m_sel = 10)**

Select m_sel variables with highest absolute standardized regression coefficients in an optimized PLS model (from X and y). Further parameters can be given for rdCV.

**sel <- varsel_stepwise_BIC (X, y, mode = “forward”, maxTime = 200, maxsteps = 20, r_step_resultfile = “r_step_BIC.RData”)**

Stepwise variable selection using the BIC criterion; mode is “forward” or forward/backward (“both”); maximum computing time and maximum number of steps can be defined, and detailed results from the selection can be stored in a result file.

**X <- Dragon60_import (dragonfile = “”, outfile = “descriptors.RData”)**

Import of Dragon 6.0 descriptor data (3 text files with basic filename dragonfile) and output to a matrix object (X) and an RData file (outfile). Missing values are converted to the *R* code NA.

**res <- rdcv_pls (X, y, sel_files = “sel.RData”, PDFfile = “rdCV_plots.PDF”)**

Performs rdCV with variable matrix X and property vector y. Parameters for rdCV (number of repetitions, number of segments, etc.) can be optionally defined. sel_files contains a logical vector for variable selection. The result object res contains e. g. all SEP values for the made repetitions, all estimations for the optimum number of PLS components, and the predicted *y*-values. The optional PDF file contains diagnostic plots.

**pls_model <- pls_one_model (X, y, a_final)**

Makes a single (final) PLS model from all *n* objects using a_final PLS components (previously optimized, e. g., by rdCV). Model parameter (regression coefficients, intercept, etc. are provided in the output object pls_model, allowing a simple application of the model to new objects.

## Summary and Conclusions

A QSPR demonstration example has been worked out using a set of partially new *R* functions for molecular descriptor data as generated by *Dragon* software. PLS was used as regression method and some basic variable selection methods have been applied. A renewed algorithm for stepwise variable selection allows the use of this method for data with up to more than 2000 variables in a reasonable computing time. A careful and cautious evaluation of the model performances allows reliable conclusions about the power of the various variable subsets.

Variable selection was considered as a separate step - not emerged from the evaluation of final models. The applied strategy *repeated double cross validation* (rdCV) for developing PLS regression models allows an estimation of the optimum number of PLS components, and independently, an estimation of the model performance for test set objects that have not been used in any step of model creation or optimization. rdCV delivers also estimations of the variation of the final optimum number of PLS components, and the final standard error of prediction (SEP), thus supporting a reasonable comparison of different variable sets. The provided software in *R* allows interested users to apply these methods without effort to other similar data sets.

There is no general rule which of the variable selection strategies is the overall best one. This also depends on the actual data set. Here we have seen that the procedure based on excluding highly correlated variables performs worse than the other procedures. This result is intuitive, because the exclusion of highly correlating variables is based on bivariate information only (correlation of one specific explanatory variable with the response), while other methods also consider the multivariate relation to the other explanatory variables. In other applications where many explanatory variables contain no relevant information for predicting the response but essentially noise, the behavior can be quite different. In that case, the noise could be influential to methods considering the multivariate associations, and bivariate techniques could be preferable.

Based on experiences with the used QSPR demonstration example and other data sets we suggest to make first an optimized PLS model with all descriptors using rdCV. These calculations give a rough estimation of the prediction performance (in the used example a mean SEP of 8.5 retention index units). Then various variable selection methods may be applied in a trial-and-error strategy, and the resulting variable subsets investigated by rdCV. In the used example the elimination of variables with a very low correlation with *y* (*R*_*XY*_^2^ < 0.1) and then a stepwise forward/backward selection resulted in a subset with 22 descriptors (out of 2688) exhibiting the best performance for new cases (mean SEP 5.6). A similar performance can be expected for a final model using this subset of variables and made from all objects.
